# Rare vertebral metastasis in a case of Hereditary Paraganglioma

**DOI:** 10.1186/1897-4287-10-12

**Published:** 2012-09-21

**Authors:** Manuel Eduardo Ribeiro da Silva, Manuel João Queiroz de Fariados Santos Carvalho, António Pedro Cacho Rodrigues, Nuno Silva Morais Neves, António Moura Gonçalves, Rui Alexandre Peixoto Pinto, Davide Carvalho

**Affiliations:** 1 Spine Group, Orthopaedic Department, Hospital São João, Porto Medical School, 4200, Porto, Portugal; 2 Endocrinology, Diabetes and Metabolism Department, Centro Hospitalar S. JoÃ£o, Porto Medical School, 4200, Porto, Portugal

**Keywords:** Familiar Paraganglioma, Vertebral Metastization, SDHB

## Abstract

Paragangliomas are rare tumours with a prevalence of 1/10000 to 1/30000. Tumors arising from the paraganglia are characteristically of low malignant potential. Vertebral metastases are exceedingly rare, and only isolated case reports have described them. The authors present the clinical course of a 47 years-old female patient with a familial paraganglioma [PGL] with vertebral metastastization, who underwent an intralesional tumor excision and corpectomy. Genetic screening demonstrated a new germinal frameshift mutation of the SDHB exon 6 [c.587-591DelC]. After surgery there was normalization of the analytical parameters and imagiologic screening. One year later she presented a new image in the the pedicle of T11 on the contralateral side of the surgical incision. She performed 2 treatments with MIBG and 1 cicle of radiotherapy that made the new lesion regress. Currently the patient does not present any clinical or analytical evidence of new metastasis.

This case outlines the clinical course of a patient with a PGL syndrome for whom a rare vertebral metastasis was diagnosed. It highlights the importance of identifying patients with germline SDHB mutations, as these patients are at a high risk of developing malignant disease.

## Background

The paragangliomas/pheochromocytomas are rare tumours with a prevalence of 1/10000 to 1/30000 [1]. The sympathetic-associated paragangliomas [PGL] arise from the adrenal medulla or from the sympathetic ganglia that extend along the paravertebral axis from the neck to the abdomen and pelvis. These tumors are usually functionally active and secrete either catecholamines or metanephrines. The parasympathetic-associated paragangliomas arise in the head and neck region and middle mediastinum, and are usually nonfunctioning. The term pheochromocytoma is commonly used for a tumor located in the adrenal gland [2].

Hereditary cases represent 35% of all pheochromocytoma and paraganglioma and are associated with gene mutations. All cases display an autosomal dominant inheritance pattern with incomplete penetrance and variable expression [3,4].

Tumors arising from the paraganglia are characteristically of low malignant potential, with only 10% developing distant spread. The great majority of metastasizing tumors has evidence of distant spread at diagnosis. Fifty percent are located at the cervical lymph nodes, and the others are evenly distributed among bone, lung and liver. Vertebral metastases are exceedingly rare, and only isolated case reports have described them [5].

The authors present the clinical course of a 47 years-old female patient with a familial paraganglioma, for whom an unusual vertebral metastasis was identified and treated.

### Case presentation

37 years-old caucasian female, without known diseases until 1997, when she complains of headaches, restless feeling, anxiety and palpitations, symptoms that she attributes to stress. Routine screening showed hypertension that was resistant to medical treatment. Laboratory analyses demonstrated an elevation in the 24 h dosage of vanilmandelic acid [VMA]: 52 mg/24 h [N: 1.4 – 6.5]; epinephrine: 21 μg/24 h [N 0–20]; norepinephrine: 3161 μg/24 h [N: 23–105]; dopamine: 711 μg/24 h [N: 65–400]; metanephrine: 91 μg/24 h [N: 52–341]; normetanephrine: 3546 μg/24 h [N 88–444]. Abdominal CT showed a 5.2 cm mass in the region of the left adrenal gland, with a corresponding hyperfixation in the MIBG scintigraphy. She underwent surgery for exploration and removal of the lesion. Intraoperatively it was found that the lesion was separated from the adrenal gland. This finding together with the pathologic report of a neoplasia invading the capsule, not going beyond it, and images of venous invasion made the diagnosis of malignant paraganglioma. Genetic screening of the patient demonstrated a new germinal frameshift mutation of the SDHB exon 6 [c.587-591DelC]. There was a family history of a cousin diagnosed at 13 years of age with a dopamine producing malignant paraganglioma, that died at 30 years of age with wide spread metastasis [cranial and lumbar].

**Figure 1:**
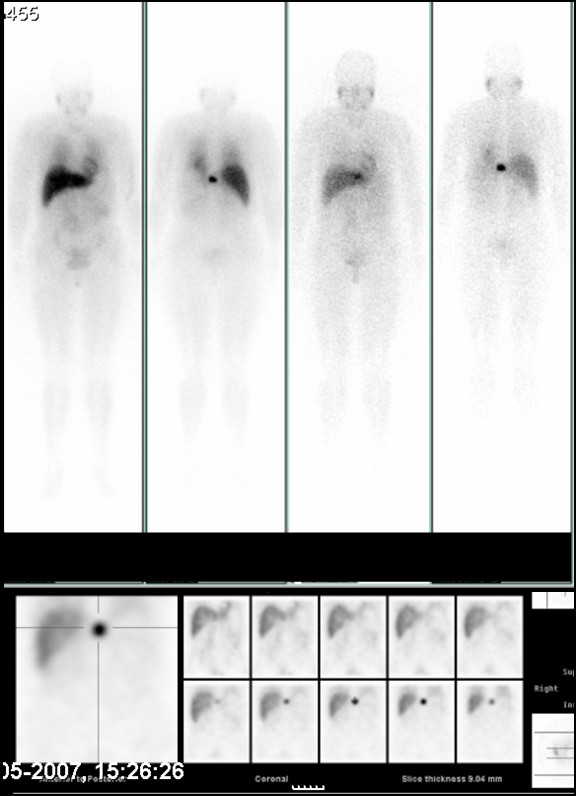
Cintigraphy revealing a solitary hyperfixation on T11.

**Figure 2:**
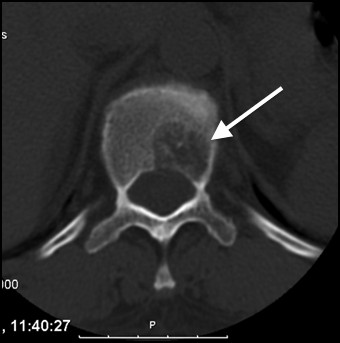
** A well delimited lytic lesion on the left side of T11 body was observed on CT scan.** This lesion conditioned a narrowing of the vertebral canal, with slight compression of the spinal cord. Arrow indicating the lesion.

**Figure 3:**
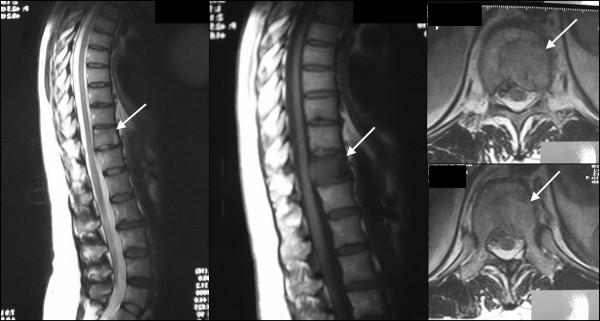
** On MRI this expansive lesion with low signal in T1 WI and high signal in T2 WI conditioned left root compression and enhanced after gadolinium injection.** These features suggest the diagnosis of a malignant lesion, most probably a metastasis. Arrow indicating the lesion.

**Figure 4:**
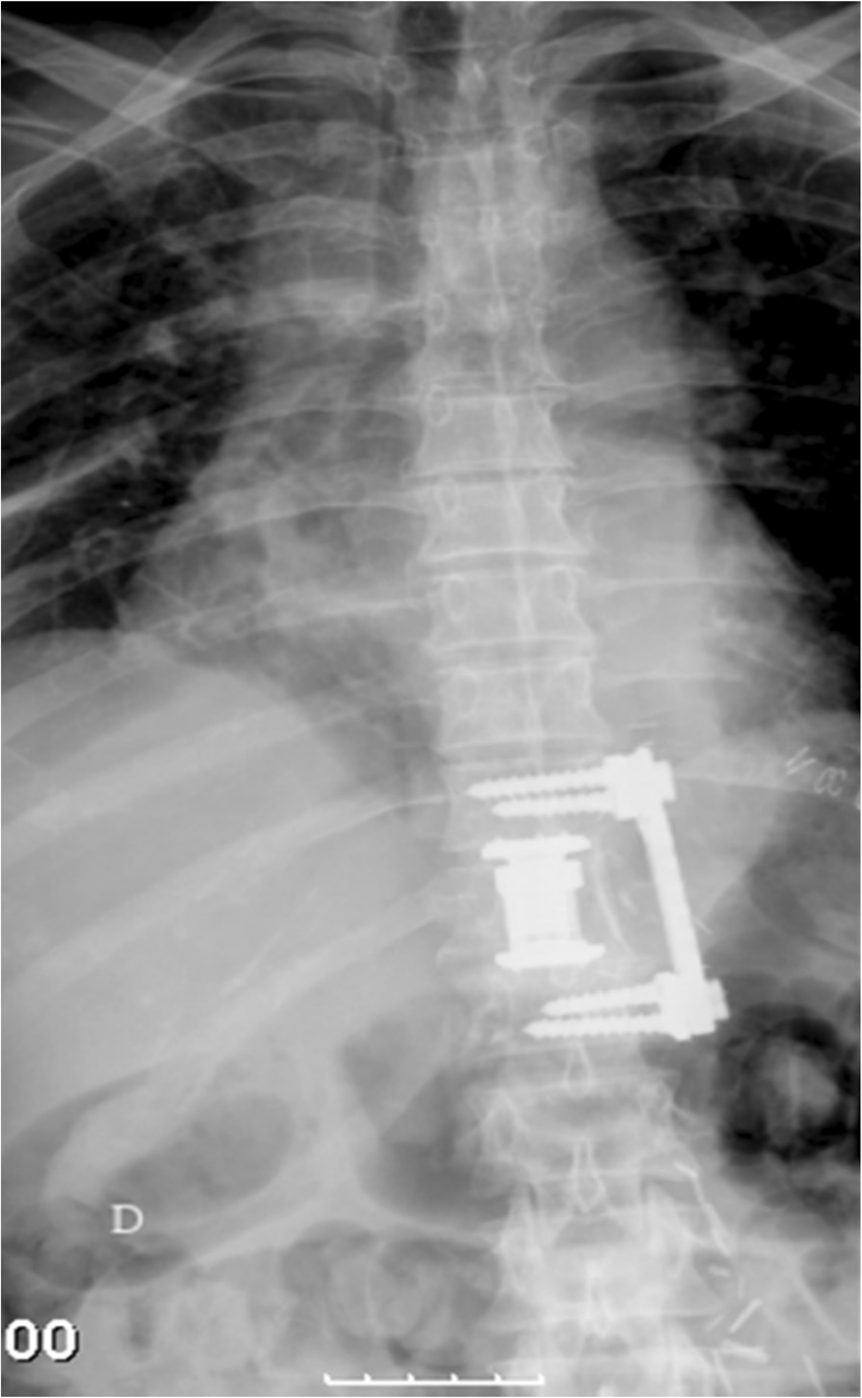
Post Op x-ray after corpectomy, replacement with an interbody spacer, autologous graft and a lateral plate.

In the post operative period there was a normalization of VMA, catecholamines and metanephrines concentrations. On March 2006 routine analyses showed an elevation of catecholamines. Abdominal MRI showed a recidiva that was confirmed by MIBG cintylogram. The patient underwent a new surgical removal of the tumor. Pathologic report confirmed this to be a local metastasis of the previous paraganglioma. Once again there was a normalization of the analytical parameters after surgery but 12 months after this episode she started complaining of back pain. Analytical evaluation showed a new elevation of VMA and catecholamines concentrations. This time scintigraphy revealed a solitary hyperfixation on T11 [Figure 1]. CT confirmed a lytic lesion on the body of T11 extending to the left pedicle [Figure 2]. On MRI this lesion presented low signal in T1 WI and high signal in T2 WI and enhanced after gadolinium injection. These features were suggestive of a probable metastasis [Figure 3]. The patient was referred to the Spine Group of our hospital and tumor removal was decided. She underwent an intralesional tumor excision and corpectomy, and replacement with an interbody spacer [Synex, Synthes], autologous graft and a lateral plate [Vantage, Medtronic] [Figure 4]. The pathologic analysis of the lesion confirmed a new recidiva of paraganglioma. After surgery there was a favorable evolution with improvement of the clinical symptoms and normalization of the analytical parameters and imagiologic screening. The patient was able to resume work. She was kept under tight medical surveillance and one year later she presented with new positive image in the lumbar region, corresponding to a new image in the pedicle of T11 in the contralateral side of the surgical incision. She performed 2 treatments with MIBG [6660 MBq [180 mCi] of MIBG 131] that made the new lesion diminish and afterwards 1 cicle of radiotherapy. The patient resumed her daily living with no limitations, and without any present clinical or analytical evidence of new metastasis.

The prevalence of catecholamines or metanephrines secreting tumors in cases of hereditary pheochromocytoma and paraganglioma disorders is 10–20% in patients with von Hippel–Lindau disease, approximately 40% in patients with multiple endocrine neoplasia type 2A and 2B, and 0.1–5.0% in patients with neurofibromatosis type 1[6,7].

The incidence of pheochromocytoma is 0.001 in the general population. There aren’t reports stating the real incidence of paragangliomas, but it is consensual that it is far bellow the incidence of pheocromocytoma. The malignant diagnosis is noted according to whether metastasis or invasion exists.

Paragangliomas are commonly bilateral and may be multicentric. Multicentricity occurs in approximately 10% of tumors. Paragangliomas characteristically have a slow and unpredictable growth pattern, making it difficult to determine the natural history of the disease [3].

There are five types of familial paraganglioma [PGL] syndromes that have been identified with mutations in the SDH genes that encode subunits of the heterotetrameric SDH complex. SDH is involved in oxidation of succinate to fumarate in the Krebs cycle and provides electrons to the mitochondrial electron transport chain [6].

Investigation has revealed that SDHD mutations [PGL type 1] are associated with multifocal head and neck paragangliomas, whereas SDHB mutations [PGL type 4] are associated with extra-adrenal disease and malignancy [8,9].

Several studies have suggested that patients who are carriers of the SDHB mutation [PGL type 4], like the patient we describe, are more likely to develop extra-adrenal lesions [abdominal or thoracic] and malignant disease [10].

The WHO defines malignant pheochromocytomas and paragangliomas as metastatic disease at sites where chromaffin tissues are not usually present. Metastatic sites include the lungs, bones, liver, and lymph nodes. Malignant tumors are typically larger, have a higher mitotic count, have extensive local or vascular invasion and express fewer peptides on immunohistochemical studies compared with benign tumors [11].

There is little correlation between histologic appearance and malignant potencial. Tumors with significant atypia and pleomorphism have often behaved in benign fashion, possibly because they were removed before metastasis. Benign-appearing tumors can behave in aggressive fashion. Several authors have found that the presence of mitosis in the pathologic specimen correlated significantly with malignancy [12]. In these series, mitotic figures were seen in all malignant tumours.

Paragangliomas often exhibit a prolonged time interval to the development of recurrence or metastasis. Recurrence and/or distant metastases have been reported to occur from 0 to 20 years after diagnosis [3,12].

In the current case the patient presented one local recidiva and a distant metastasis 9 and 10 years after the initial diagnosis, respectively. In both situations it was decided that the patient was eligible for surgical treatment with curative intention because these were always isolated lesions at the time of diagnosis [13].

The patient underwent a biannual monitoring program with physical examination and measurement of blood pressure and levels of urinary catecholamines and metanephrines [14]. The fact that the patient presented only a solitary metastasis was the reason the authors chose to pursuit a surgical exertion, in an attempt to perform a curative treatment. It was taken under consideration the fact that metastatic paraganglioma, like pheochromocytoma, is relatively radioresistant, as compared with lymphoma and breast cancer [13].

The reason to choose a surgical intervention instead of chemotherapy in the T11 lesion was also based in the risk of vertebral colapse with the consequent danger of neurological injury [15]. The exertion of the lesion and the replacement by a titanium cage and ilac gaft was considered to be a better mechanical solution for the patient, besides the fact that we intended to have a curative procedure. Nevertheless a new recidiva was diagnosed one year later. An en bloc excision would probably have been more appropriate, but at the time we were not performing this routinely.

Nevertheless, this proved to be at least partially a correct decision because the patient became assymptomatic and was able to resume working [she is still working and with no clinical complaints].

One could argue that she should have had chemotherapy with MIBG imediately after the removal of the vertebral metastasis because it was the second recidiva, but it was decided against that in group reunion. It was taken in consideration that chemotherapy does not seem to increase the survival in women with metastatic malignant paraganglioma [16].

When the local vertebral recidiva occurred it was decided that the patient would undergo MIBG treatment. This option led to a regression in tumour size. Even in this perpective the surgical procedure was benefic to the patient because the dose of MIBG used is size dependent.

The patient's direct family was offered genetic screening, having been diagnosed 5 positive patients, and one already died of metastatic pheocromocytoma [17].

## Conclusions

This case outlines the clinical course of a patient with a PGL type 4 syndrome for whom a rare vertebral metastasis was diagnosed. The case highlights the importance of offering targeted genetic testing for the SDHB gene mutations to a patient with multiple, extra-adrenal paragangliomas and a family history of malignant paraganglioma. It also illustrates the importance of identifying patients with germline SDHB mutations, as these patients are at a high risk of developing malignant disease. On the other hand it also highlights the importance of a multidisciplinary approach to these patients, as well as an aggressive attitude towards metastasis, with radical surgical exertion of the tumor being, whenever it is possible, the authors’ choice.

### Consent

Written informed consent was obtained from the patient for publication of this Case report and any accompanying images. A copy of the written consent is available for review by the Editor-in-Chief of this journal.

## Competing interests

The authors did not receive any outside funding or grants in support of their research for or preparation of this work. Neither they nor a member of their immediate families received payments or other benefits or a commitment or agreement to provide such benefits from a commercial entity. The author(s) declare that they have no competing interests'.

## Authors’ contributions

MRS: acquisition of data, data analysis and manuscript drafting, MSC: acquisition of data, data analysis and manuscript drafting, PCR: acquisition of data, data analysis, NN: analysis and interpretation of data, revising, AMG: analysis and interpretation of data, revising, RP: analysis and interpretation of data, revising, DC acquisition of data, analysis and interpretation of data, revising. All authors read and approved the final manuscript.
